# Sleep-dependent motor memory consolidation in older adults depends on task demands

**DOI:** 10.1016/j.neurobiolaging.2014.12.014

**Published:** 2015-03

**Authors:** Christel Gudberg, Katharina Wulff, Heidi Johansen-Berg

**Affiliations:** a Oxford Centre for Functional MRI of the Brain (FMRIB), Nuffield Department of Clinical Neurosciences, University of Oxford, John Radcliffe Hospital, Oxford, UK; b Nuffield Laboratory of Ophthalmology, Nuffield Department of Clinical Neurosciences, University of Oxford, John Radcliffe Hospital, Oxford, UK

**Keywords:** Sleep, Motor memory, Consolidation, Aging, Fine motor skill, Sequence learning

## Abstract

It is often suggested that sleep-dependent consolidation of motor learning is impaired in older adults. The current study challenges this view and suggests that the degree of motor consolidation seen with sleep in older age groups depends on the kinematic demands of the task. We show that, when tested with a classic sequence learning task, requiring individuated finger movements, older adults did not show sleep-dependent consolidation. By contrast, when tested with an adapted sequence learning task, in which movements were performed with the whole hand, sleep-dependent motor improvement was observed in older adults. We suggest that age-related decline in fine motor dexterity may in part be responsible for the previously described deficit in sleep-dependent motor consolidation with aging.

## 1 Introduction

The formation of memories in humans is underpinned by highly specialized processes of encoding, consolidation, and retention. Initially labile new memory traces undergo postencoding processing, which aids in stabilizing and integrating learned material over time (Diekelmann et al., 2009; Rasch and Born, 2013; Stickgold, 2009) and frequently enables further postlearning improvements associated with off-line consolidation (Doyon et al., 2009; Robertson et al., 2004; Trempe and Proteau, 2010). Depending on the type of material being learned, these off-line gains may occur during wakefulness and/or during sleep.

After a single session of learning a novel motor sequence, healthy young adults consistently show off-line gains in performance and in the case of explicit sequence learning particularly after an off-line period of sleep (Fischer et al., 2002; Robertson et al., 2004; Walker et al., 2002, 2003). By contrast, a growing number of studies have found that such improvements, immediately after a period of sleep, are lacking in healthy older adults (Fogel et al., 2013; Spencer et al., 2007; Wilson et al., 2012). Tucker et al. (2011) found a decline in performance of a motor sequence in older adults after a 12-hour period of wakefulness, in contrast to maintained performance after a 24-hour period containing both sleep and wake. Although this interesting result could be interpreted as consistent with the possibility of consolidation during sleep, the authors did not find improvements in performance after sleep but rather just a smaller decrement in performance. In addition, the design did not control for the passage of time (24 hours in the sleep condition compared with 12 hours in the wake condition), and so it remains unclear whether sleep-dependent consolidation of motor sequence learning occurs in older adults.

Multiple factors may contribute to this age-related discrepancy. It is well established that sleep architecture changes with age (e.g., Colrain et al., 2010; Crowley et al., 2002; Mander et al., 2013; for meta-analysis see Ohayon et al., 2004). Studies in younger adults have shown significant associations between specific sleep characteristics (e.g., sleep spindle and slow-wave activity) and motor consolidation (Huber et al., 2004; Landsness et al., 2009). It is therefore possible that age-related changes to sleep architecture and activity contribute to a reduced capacity for consolidation of motor learning during sleep (Fogel et al., 2013; King et al., 2013).

However, another issue that has been overlooked previously is the degree to which decline in movement dexterity may contribute to observed differences. There is evidence to suggest significant reductions in fine motor skill, including speed, dexterity, and finger strength with older age (Ashendorf et al., 2009; Dayanidhi and Valero-Cuevas, 2014; Marmon et al., 2011; Ranganathan et al., 2001; Soer et al., 2012). Such effects are thought to partly reflect age-related changes in cortical inhibitory processes important for fine motor performance (e.g., by suppressing coactivation of agonist and antagonist muscles; Heise et al., 2013; Klass et al., 2007; Marneweck et al., 2011). The sequence learning tasks that are typically used to assess sleep-dependent motor consolidation require rapid, individuated finger movements. Therefore, it is possible that age-related changes in fine motor dexterity impact on performance during training, which in turn could influence off-line consolidation in older adults. However, one previous study that required older adults to perform an explicit sequence-tracking task using a hand-operated joystick, which would not require individual finger movements, did not find clear evidence for sleep-dependent consolidation (Siengsukon and Boyd, 2009). Nevertheless, in contrast to the evidence on consolidation of fine motor tasks, which tend to show a lack of improvement in performance immediately after sleep in older adults (Fogel et al., 2013; Spencer et al., 2007; Wilson et al., 2012), the joystick tracking task did produce improvements in performance after sleep in older adults, but these did not differ significantly from the gains seen after a comparable period of wakefulness. It is possible that individual task demands may influence the degree to which consolidation of motor learning after sleep can be detected in older adults.

In summary, existing studies do not provide clear evidence for sleep-dependent consolidation of motor learning in older adults and have not directly addressed whether the presence of consolidation depends on task demands. To address these questions, we tested off-line consolidation of motor learning in both younger and older adults by using either a classic version of the motor sequence task, requiring individual finger movements, or an adapted version of the same task, using whole hand movements.

## 2 Methods

### 2.1 Participants

A total of 49 younger (aged 18–35) and 42 older (50–85) healthy, right-handed participants provided written informed consent to participate in accordance with local ethics committee guidelines. Participants were assigned pseudorandomly to different experimental condition groups (Table 1). Participants had no previous history of neurologic, psychiatric, or sleep disorders or drug or alcohol abuse, and they were instructed to remain free of caffeine, alcohol, and drugs (apart from prescribed medication not expected to have an influence on sleep quality, such as for blood pressure, birth control, and nondrowsy antihistamines) for the duration of the study, as well as for 12 hours before taking part. Participants also were instructed to refrain from napping during the day, confirmed verbally at the relevant posttraining retest session. One participant reported having a nap after initial training, and 1 participant consistently reproduced only the first 4 digits of the number sequence at retest. Behavioral consolidation data from these 2 participants were therefore excluded from further analysis.

Nine participants (from the older groups) took part in 2 conditions. In these cases, different conditions were counterbalanced to control for order effects and tested at least 1 month apart, and different sequences with completely unique grammars were used for each condition. In these circumstances, we would not expect any effect of the earlier condition on the later condition (Walker et al., 2003). However, to guard against the possibility that results from these participants were having a disproportionate effect on our findings we also redid any relevant analyses without data from the second condition of these participants, with very similar results (these are provided in the Supplementary Materials).

### 2.2 Sequence learning tasks

**Fig. 1:**
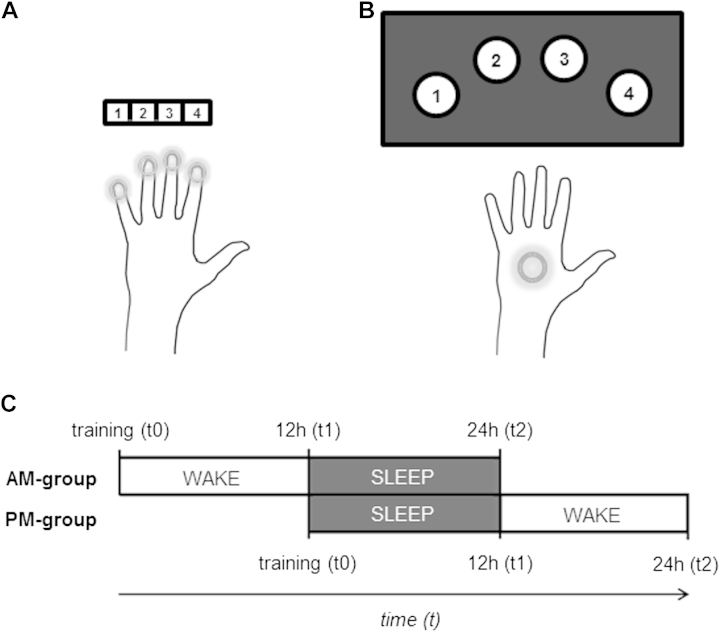
Task design and setup. Participants performed the 5-digit sequence with their nondominant (left) hand either for the classic (A) or adapted (B) versions of the sequence learning task. Participants were pseudorandomly assigned to receive their initial training (t0) either in the morning (AM group) or in the evening (PM group), with retests 12 (t1) and 24 (t2) hours after training (C).

Depending on the group to which participants were assigned, they performed either a standard sequence learning task (classic; Fig. 1A; Walker et al., 2002, 2003) or an adapted whole-hand sequence task (adapted; Fig. 1B). Tasks were matched on all attributes apart from requiring either fine finger or whole hand movements. For the classic task, button presses were made with the index, middle, ring, and little fingers of the (nondominant) left hand on a standard computer keyboard. For the adapted task, button presses were performed with the (nondominant) left hand, with buttons spaced 22° apart and positioned along a curve with a radius of 27.26 cm (equal to the average adult forearm length; Plagenhoef et al., 1983) to allow comfortable reach of all buttons while keeping the left elbow positioned on a padded mat on the table. A 5-digit numeric sequence (e.g., 4-1-3-2-4) was presented on the screen during the entire period participants performed the sequence to prevent any working memory requirement. To avoid providing accuracy feedback, responses elicited only a white dot, which moved from left to right in accordance with the number pressed to indicate the response had been recorded. Participants were instructed to repeat the sequence as fast and as accurately as possible for 30 seconds followed by a 30-second rest period. Each participant performed 12 blocks (sequence + rest) during training (t0) lasting 12 minutes in total. At the first retest session (t1), participants performed only 2 consecutive blocks to reduce any influence of additional practice or training between retest sessions.

### 2.3 Procedure

Training (t0) took place between 8:00 and 10:30 AM (for the AM group) or 8:00 and 10:30 PM (for the PM group). The 2 retest sessions took place 12 (t1) and 24 (t2) hours after training (Fig. 1C). For morning sessions, testing took place at least 1 hour after participants woke up. Before the start of each session, participants completed the Stanford Sleepiness Scale to indicate their level of subjective alertness (Hoddes et al., 1973). For the 24-hour period of study participation, participants wore an activity monitor on their nondominant wrist (digital accelerometer; Actiwatch-Light; CamNtech Ltd, Cambridge, UK) and were asked to keep an activity log, which together were used to provide measures of sleep-wake patterns (Rogers et al., 1993; Sadeh and Acebo, 2002). Participants also completed the Pittsburgh Sleep Quality Index (PSQI), which is a measure of self-reported sleep quality over the previous month (Buysse et al., 1989).

### 2.4 Behavioral measures

*Performance rate* (number of correct complete sequences per block) was used as the main behavioral measure, as in previous studies of motor consolidation (Karni et al., 1995). *Difference scores* were calculated for the 2 consolidation periods (sleep/wake) as in previous work (e.g., Nishida and Walker, 2007; Spencer et al., 2007; Wilson et al., 2012). For the first consolidation period, this score was the difference in performance rate from the end of training (t0_blocks11,12_, average of final 2 blocks of training at t0) to the first retest session (t1_blocks1,2_, average of the 2 blocks performed at t1 [when only 2 blocks are performed in total]), divided by t0_blocks11,12._ Similarly, for the second consolidation period, this score was calculated from the first retest to the second retest as (t2_blocks1,2_ − t1_blocks1,2_)/t1_blocks1,2_. For the AM group, the t0-t1 interval consisted of wakefulness and the t1-t2 interval consisted of sleep; the order of the intervals was reversed for the PM-group (Fig. 1C). *Actigraphy recordings* were used to derive sleep-wake patterns, including indirect measures of sleep quality and quantity, and were scored with manual editing based on the activity logs and an automated scoring algorithm using Sleep Analysis v7.23 software (CamNtech Ltd). *PSQI questionnaire* data were scored manually based on scoring criteria outlined by Buysse et al. (1989). A greater global PSQI score is associated with poorer self-reported sleep (range 0–21) with a score greater than 5 suggesting poor sleep, as validated against clinical and laboratory measures (Buysse et al., 1989).

### 2.5 Statistical analysis

Primary statistical analyses are based on repeated measures analysis of variance (ANOVA) with within-group factors of performance rate per block for the analyses on in-session training, and sleep versus wake for the analyses on off-line consolidation, and between-subject factors of age group (younger, older), task (classic, adapted) and training time (AM, PM), and were followed up with ANOVAs split by age group and/or task to test for task- and age-specific effects. For ANOVAs split by age group, participant age also was included as a covariate to control for age variation within the group. One-sample *t*-tests were used to compare performance change after consolidation periods (t1-t0/t0, t2-t1/t1) against zero. Independent samples *t*-tests and one-way ANOVAs were used to compare sleep quality measures between age and task groups. Pearson's coefficient was adopted for all correlational analyses.

## 3 Results

### 3.1 Sleep quality measures

We first tested whether our indirect measures of sleep quality (i.e., questionnaire data, actigraphy measurements, activity logs) differed between age groups or correlated with behavioral measures. Self-reported sleep quality, as assessed by the global PSQI score, was significantly poorer in the older group compared with the younger group (t[78] = 2.27, *p* = 0.026). However, although actigraphy revealed that the older group woke earlier (F[1,52] = 21.62, *p* < 0.001) and got up earlier (F[1,52] = 24.36, *p* < 0.001) than the younger controls (Table 2), no significant differences in the main measures of sleep quality were observed between age groups (including actual sleep time, sleep efficiency, and sleep latency). No other significant differences in sleep measures were found between age groups. Moreover, no correlations were found between any of the subjective or objective sleep quality measures and the difference scores after sleep, either when we pooled across participants or when we considered each age group and task separately. Therefore, given the observed difference between age groups in subjective sleep quality (as reflected in the significant difference in global PSQI scores) but not in objective sleep quality (as reflected in the lack of age effect for the main actigraphy measures), we have covaried out any effect related to subjective sleep quality in the relevant analyses of consolidation.

We also wanted to test whether any differences in consolidation effects observed between the 2 tasks could be due to variability in (objective) sleep quality or quantity specifically between task groups (Table 2). A one-way ANOVA showed that task groups (classic, adapted) did not differ significantly on the primary measures of sleep during the consolidation night (actual sleep time, F[1,52] = 0.09, *p* = 0.77; sleep efficiency, F[1,52] = 0.22, *p* = 0.64; sleep latency, F[1,52] = 0.002, *p* = 0.96). No other significant differences in sleep measures were found between task groups, either across participants or when considering age groups separately.

### 3.2 Behavioral performance during initial training

**Fig. 2:**
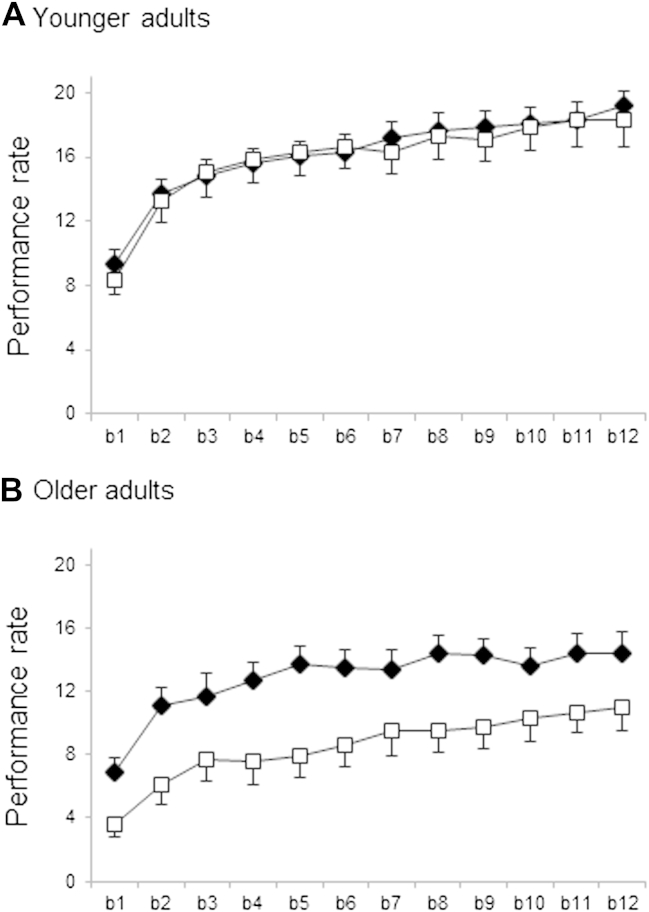
Learning curves in younger and older participants. Mean number of correct sequences performed per block (b_k_) was our measure of performance rate during the training session (classic task: open squares and adapted task: filled diamonds). Whereas the younger group (A) showed very similar performance across the 2 tasks, the older group (B) showed markedly poorer performance on the classic compared with the adapted task. Fig. S1 (provided in Supplementary Materials) depicts learning curves and performances after the off-line periods of consolidation (t1, t2) for each of the individual training times (AM, PM) and for each age group (younger, older) and task (classic, adapted).

Performance rates during the initial training session for each experimental group are plotted in Fig. 2 (detailed plots for each of the age, task, and training groups are included in the Supplementary Materials). To investigate changes in performance across blocks during training, we performed a repeated measures ANOVA on within-session values for performance rate per block with between-subject factors of age group (younger, older), training time (AM, PM), and task (classic, adapted), and the within-subject factor of block (blocks 1–12). Results revealed significant main effects of block (F[7.65,634.60] = 117.61, *p* < 0.001), age group (F[1,83] = 47.62, *p* < 0.001) and task (F[1,83] = 8.66, *p* < 0.005). These main effects reflected improving performance over blocks, and better performance on average in the younger group, and with the adapted task. In addition, we found significant interactions of block by age group (F[7.65,634.60] = 2.24, *p* < 0.05), task by age group (F([1,83] = 6.79, *p* < 0.05), and a significant 3-way interaction of task × age group × time of day (F[1,83] = 4.55, *p* < 0.05).

Given these interactions with age group, we followed up with repeated measures ANOVAs split by age group. Despite the interaction between age group and block in the initial ANOVA, we found trends for significant main effects of block in both younger (F[7.49,322.03] = 1.87, *p* = 0.07) and older participants (F[5.49,192.08] = 2.00, *p* = 0.07), suggesting that performance improved across training in both age groups. However, consistent with the significant interaction between task and age group in the initial ANOVA, we found a significant main effect of task for older (F[1,35] = 12.87, *p* < 0.005) but not for younger participants (F[1,43] = 0.22, *p* = 0.64). This reflected markedly reduced performance on the classic task, compared with the adapted task, in the older group only (Fig. 2). However, overall change in performance during training (i.e., [mean of blocks 11,12] − [mean of blocks 1,2]) was comparable between the 2 tasks in the older group (mean change in # correct sequences: 5.33 ± 0.57 SEM, adapted; 5.93 ± 0.52 SEM, classic; t(40) = -0.77, *p* = 0.45). When comparing across age groups, we found no significant effect of age on learning during the initial training session on the classic sequence task but found significantly reduced learning in the older group for the adapted task (classic: 7.48 ± 0.71 SEM, younger; 5.93 ± 0.52 SEM, older; t[42] = 1.73, *p* = 0.09; adapted: 7.23 ± 0.51 SEM, younger; 5.33 ± 0.57 SEM, older; t[45] = 2.49, *p* = 0.017).

To test for the possibility of time of day effects in initial training profiles, we compared performances between the AM and PM groups at baseline (mean of blocks 1,2) and end of training (mean of blocks 11,12), taking into account task and age group but found no evidence for differences between groups (F[1,87] = 0.96, *p* = 0.33; mean baseline: 8.77 ± 3.89 SD, AM group; 9.60 ± 4.40 SD, PM group; mean final performance: 15.61 ± 5.06 SD, AM group; 15.87 ± 5.49 SD, PM group).

### 3.3 Off-line effects across groups

**Fig. 3:**
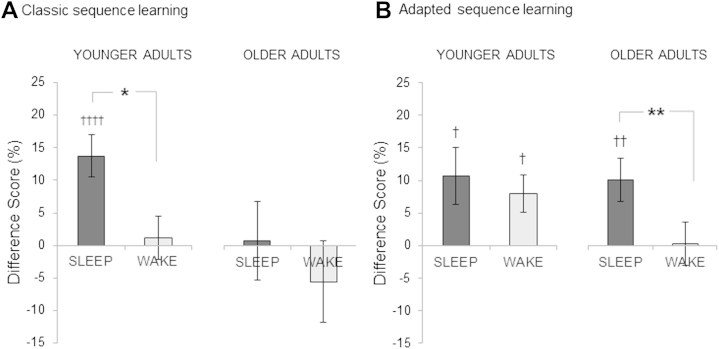
Summary of consolidation effects across groups. Bar chart (A) shows significant improvements in performance only after sleep, for younger but not older adults on the classic task. In contrast, following a night of sleep after training on the adapted task (B), older adults show significant sleep-dependent improvements. * = *p* < 0.05, ** = *p* < 0.01 main effect of sleep versus wake; † = *p* < 0.05, †† = *p* < 0.01, †††† = *p* < 0.001, one-sample *t*-test versus zero.

Difference scores after sleep or wakefulness, averaged by group and task, are plotted in Fig. 3. To test whether sleep significantly benefits task performance over and above the simple passage of time, and whether this effect is modulated by age, we ran a repeated measures ANOVA on the difference scores, including a within-subject factor indicating whether the period included sleep or wakefulness (sleep, wake), as well as between-subject factors of age group (younger, older), task (classic, adapted), and training time (AM, PM). Results showed significant main effects of sleep versus wake (F[1,81] = 5.83, *p* = 0.018), age group (F[1,81] = 11.01, *p* = 0.001) and task (F[1,81] = 4.78, *p* = 0.032). These reflected overall greater off-line improvements in performance with sleep, in the younger group, and with the classic task. We also found a significant interaction between sleep versus wake and training time (F[1,81] = 6.39, *p* = 0.013), as well as significant 3- and 4-way interactions (sleep vs. wake × age group × training time, F[1,81] = 7.75, *p* = 0.007; sleep vs. wake × age group × task × training time, F[1,81] = 5.78, *p* = 0.019).

To further follow up on these interactions we ran additional ANOVAs split by task and age group.

### 3.4 Off-line effects–classic task

In line with previous research in younger groups, we found significant sleep-dependent consolidation in younger adults (sleep vs. wake, F[1,17] = 6.00, *p* = 0.025), with significant improvements for this group found during sleep (one-sample *t*-test: t[21] = 4.28, *p* < 0.001) but not wakefulness (t[21] = 0.34, *p* = 0.74; Fig. 3A), as well as a significant interaction with age within the younger groups (F[1,17] = 6.98, *p* = 0.017). By contrast, we found no evidence for sleep-dependent consolidation in older adults (sleep vs. wake, F[1,11] = 1.42, *p* = 0.26; Fig. 3A), and found no significant improvements in performance for this group after either sleep (t[19] = 0.12, *p* = 0.91) or an equivalent period of wakefulness (t[19] = −0.88, *p* = 0.39). No other main effects or interactions were found.

There were no significant effects of training time (i.e., AM or PM) on difference scores for either group (younger, F[1,17] = 0.028, *p* = 0.87; older, F[1,11] = 0.15, *p* = 0.71), but we did find a significant interaction between training time of day and difference scores (sleep, wake) in older adults (F[1,11] = 8.17, *p* = 0.016), that was not present in the younger adults (F[1,17] = 1.40, *p* = 0.25). This reflected greater gains after sleep compared to wake for older adults who trained in the morning (AM group). By contrast, older adults who trained in the evening (PM group) showed greater improvements after wake compared with after sleep. No other main effects or interactions were found.

### 3.5 Off-line effects–adapted task

In contrast to the findings for the classic task, we did find evidence for sleep-dependent consolidation in the older adults with the adapted task (Fig. 3B), reflected in a significant main effect of sleep versus wake (F[1,15] = 8.55, *p* = 0.01), with significant improvements found after sleep (t[20] = 3.03, *p* = 0.007) but not wakefulness (t[20] = 0.07, *p* = 0.95), as well as a significant interaction with age (F[1,15] = 6.06, *p* = 0.026) within the older groups. No other main effects or interactions were found.

In younger adults, there was no evidence for sleep-dependent effects with the adapted task (sleep vs. wake, F[1,20] = 0.54, *p* = 0.47), with significant improvements found during both sleep (t[25] = 2.46, *p* = 0.02) and wakefulness (t[25] = 2.76, *p* = 0.01; Fig. 3B), as well as a significant main effect of training time of day for the younger adults (F[1,20] = 5.03, *p* = 0.036). No other main effects or interactions were found.

### 3.6 Correlations between on-line and off-line gains in performance

**Fig. 4:**
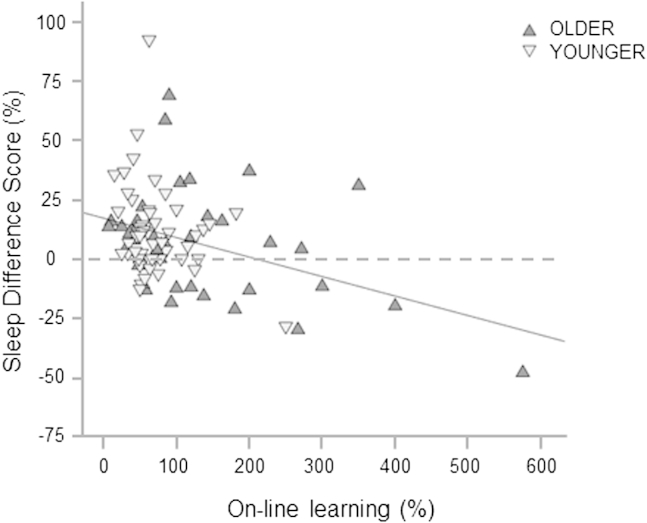
Correlation between on-line and off-line changes across groups. On-line improvement is relative to baseline performance (i.e., mean of blocks 1, 2). Dotted line signifies threshold for consolidation (improvement) with sleep, below which performances worsened overnight.

We went on to test for relationships between learning during initial training and subsequent off-line consolidation effects. Across all participants we found a significant correlation between difference scores after sleep and overall learning in-session (r = −0.36, *p* < 0.001), such that the more on-line learning that took place during training, the less off-line consolidation was shown to occur with sleep (Fig. 4). No correlation was found between change in-session and following an equivalent period of wakefulness (r = 0.07, *p* = 0.51). These 2 correlations differed significantly using Fisher's *r*-to-*z* transformation (z = −2.9, *p* = 0.004).

## 4 Discussion

These findings provide, to our knowledge, the first evidence of sleep-dependent consolidation of motor learning in older adults. We found a clear dissociation of consolidation effects between 2 versions of a motor sequence task and suggest that previously reported deficits in consolidation in older adults may be attributable to age-related differences in fine motor skill affecting the acquisition phase of fine motor learning. Our findings reveal strong sleep-dependent consolidation effects in older adults when kinematic constraints related to fine finger movement are removed. Specifically, we show that although older adults performing the classic sequence learning task (requiring fine motor control of individual fingers) do not show sleep-dependent consolidation effects, older adults performing the kinematically adapted task (using whole hand rather than individual finger movements) show sleep-dependent consolidation.

The finding of sleep-dependent consolidation of motor learning in older adults contrasts with previous studies reporting absent or reduced consolidation during sleep in older adults (Fogel et al., 2013; Spencer et al., 2007; Tucker et al., 2011; Wilson et al., 2012). Although Tucker et al. (2011) found a notable effect of delayed learning in their older group, there was no improvement in performance at immediate retest after the consolidation period. Importantly, these previous studies have used sequence learning tasks requiring individual finger movements, on which we also found no evidence for consolidation during sleep in older adults. Such tasks require fine motor skill and there is growing evidence that this ability significantly diminishes with age (Ashendorf et al., 2009; Dayanidhi and Valero-Cuevas, 2014; Marmon et al., 2011; Ranganathan et al., 2001; Soer et al., 2012). This decline may arise in part due to peripheral changes (Klass et al., 2007) and in part as a result of changes in brain morphometry (Good et al., 2001; Lemaitre et al., 2005, 2012; Raz et al., 1997; Salat et al., 2004; Smith et al., 2007) within regions that have been shown to play a key role in fine motor function and, particularly, in sequence learning (Mattay et al., 2002; Orban et al., 2011; Voineskos et al., 2012).

Consistent with these findings, our results reveal significant behavioral differences in older adults depending on fine motor requirement. Specifically, we show that although significant learning (i.e., increase in performance rate across training blocks) was found for both sequence learning tasks in older adults, overall performance (i.e., mean performance rate over all training blocks) was reduced significantly on the classic sequence task (requiring fine motor movement) compared with the adapted task, in older adults (Fig. 2B). We found that, when the requirement for fine motor ability was removed, as in the adapted task, significant sleep-dependent consolidation was observed.

Our findings predict that other sequence learning tasks that do not rely on fine motor skill will reveal similar sleep-dependent consolidation in older adults. However, Siengsukon and Boyd (2009), using a joystick tracking task, found only a nonsignificant improvement after sleep (and wake), and so does not support the prediction that all tasks with low-dexterity demands show sleep-dependent consolidation in older adults. Future work is needed to explore whether task parameters (kinematic constraints, explicit vs implicit learning strategies) influence the degree to which sleep-dependent consolidation is seen in older adults.

We have focused on the presence of sleep-dependent effects in older adults observed for the adapted task and not for the classic task. However, it is also notable that the younger adults show off-line gains in performance during both wake and sleep for the adapted task (Fig. 3B). Therefore, an alternative interpretation of our pattern of results is that, compared with younger adults, older adults fail to show off-line improvement during wake. It is somewhat surprising to see significant consolidation during wake for the younger adults performing the adapted task, as this is not present for the classic task here. However, although previous studies using the classic task have similarly not reported significant consolidation during periods of wake, modest improvements in average performance (typically 20%–40% of the improvement seen during sleep) have often been found (e.g., Nishida and Walker, 2007; Walker et al., 2002, 2003). Therefore, it is possible that consolidation during wake can be observed for an explicit sequence learning task, albeit typically to a lower degree than consolidation during sleep. Future studies should test whether such effects are more pronounced using the adapted task, as suggested by our results in younger adults.

In addition to our main finding that sleep-dependent consolidation can be observed in older adults when kinematic demands are relaxed, we also found evidence that older adults may benefit from an extended period of off-line consolidation for the classic version of the sequence learning task. This observation arises from the finding that older adults who trained in the morning (AM group) showed greater gains after sleep (24 hours later) compared with wake (12 hours later), whereas older adults who trained in the evening (PM group) showed greater improvements after wake (24 hours later) compared with after sleep (12 hours later). This finding is partially consistent with previous research showing that an extended consolidation period (24 hours) could facilitate performance maintenance in older adults (Tucker et al., 2011).

To rule out the possibility that circadian fluctuations affected performance levels, we controlled for time of day effects across task and age groups. We found no significant differences in performance between participants who trained in the morning (AM group) or the evening (PM group). Moreover, we did not find significant differences in subjective ratings of alertness between sessions that took place at different times of the day, consistent with previous reports (e.g., Walker et al., 2003). Therefore, it seems unlikely that the effects observed here could be explained by circadian fluctuations alone.

Considering behavioural data from the initial training sessions, we found significant learning (i.e., improvement in performance rate from the first 2 to the last 2 blocks of the initial training session) for both age groups and both tasks (Fig. 2). Similar to previous work (e.g., Spencer et al., 2007; Tucker et al., 2011; Wilson et al., 2012), we find broadly comparable learning scores between younger and older adults for the classic task. However, we did find reduced initial learning in the older adults for the adapted task, for which the effects of age have not been assessed previously.

Our finding of age-related differences in sleep behavior based on actigraphy and self-reported measures is consistent with previous literature (Bagai et al., 2013; Moser et al., 2009; Yoon et al., 2003). These measures were highly comparable between task groups, suggesting that observed differences in consolidation effects between the classic and adapted sequence tasks are unlikely to be due to variability in sleep quality or quantity between task groups. However, no correlations were found between sleep measures and difference scores. It is possible that a richer measure of sleep, such as that provided by electroencephalography, would be more sensitive to detect correlations between sleep characteristics and consolidation, as shown previously for younger adults (e.g., Huber et al., 2004). Although the concordance between actigraphy and polysomnography are reported to be greater than 85% in different healthy age groups (Acebo and LeBourgeois, 2006), actigraphy may have limited ability to detect wake states without sufficient movement (Paquet et al., 2007; de Souza et al., 2003), leading to inaccurate measures of sleep. However, the use of manual editing, informed by the activity logs, is likely to have improved the resulting accuracy, consistent with previous reports (Yoon et al., 2003). It should also be noted that actigraphy recordings only took place for the 24 hours participants were involved in the study. Although this provides us with an estimate of sleep for the specific night that is relevant to the behavioral tests, it would not be expected to provide an accurate measure of habitual sleep quality. Indeed, similar to previous work (e.g., Grandner et al., 2006) we found no significant relationship between our short-term recordings of actigraphy and PSQI, which asks about longer-term sleep habits.

In summary, we provide evidence for sleep-dependent consolidation of motor learning in older adults. We show that, by removing the demand on fine motor skill placed by most traditional sequence tasks, sleep-specific consolidation can be revealed also in older adults. We further show that off-line gains can be observed on the classic sequence learning task, but that, for this version of the task, older adults benefit more from extended periods of consolidation than specifically from sleep alone. These findings have implications for future studies of consolidation in aging, including dissociating specific mechanisms of sleep that underpin the consolidation process in older adults and, importantly, whether these mechanisms are similar to, or significantly diverge from, those reported in healthy younger adults.

## Disclosure statement

The authors have no actual or potential conflicts of interest.

## Figures and Tables

**Supplementary Fig. 1:**
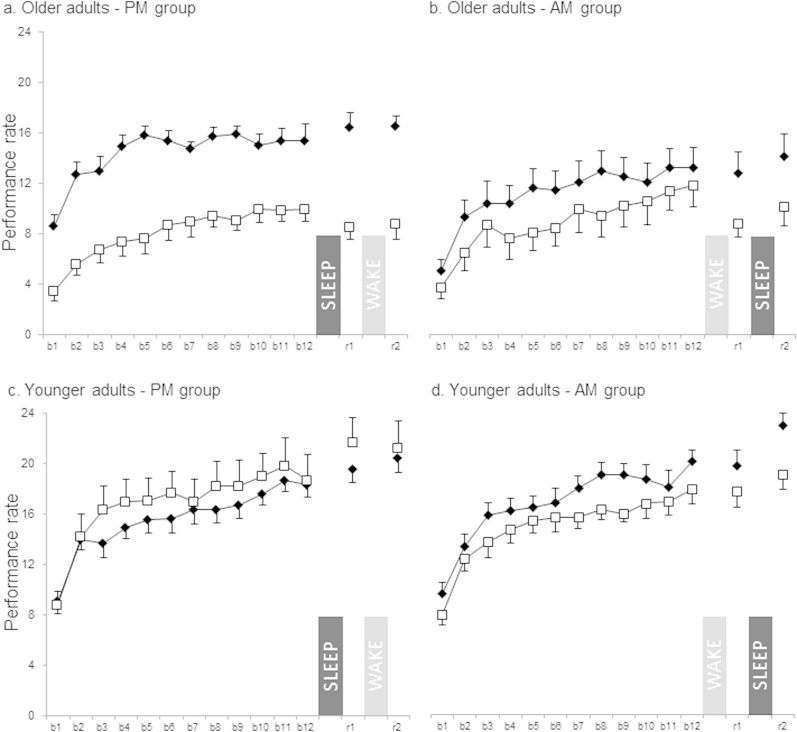
Shows performance levels for individual age and task groups for both AM and PM training times. Classic task: open squares and adapted task: filled diamonds.

**Table 1:** Participant details

Task (by age group)	Mean age (±SEM)	n	Training group
Younger adults			
Classic	24.50 (±0.89)	13	AM
Classic	24.40 (±0.82)	10	PM
Adapted	24.31 (±0.94)	13	AM
Adapted	25.46 (±0.95)	13	PM
Older adults			
Classic	67.22 (±3.19)	10	AM
Classic	67.90 (±2.99)	11	PM
Adapted	66.30 (±2.77)	10	AM
Adapted	65.18 (±3.22)	11	PM

Key: AM, participants trained in the morning; PM, participants trained in the evening; SEM, standard error of the mean.

**Table 2:** Actigraphy and PSQI measures across tasks and age groups

Sleep measures	Younger adults	Older adults	Classic task	Adapted task
Actigraphy (hh:mm)				
Bed time	23:44 ± 00:09	23:23 ± 00:10	23:37 ± 00:10	23:34 ± 00:10
Sleep start	00:01 ± 00:09	23:35 ± 00:10	23:52 ± 00:08	23:49 ± 00:12
Sleep end	07:30 ± 00:07	06:33 ± 00:09	07:06 ± 00:10	07:09 ± 00:09
Get up time	07:36 ± 00:06	06:44 ± 00:08	07:17 ± 00:08	07:13 ± 00:08
Actual sleep time	06:24 ± 00:09	06:11 ± 00:11	06:21 ± 00:09	06:16 ± 00:10
Sleep latency	00:17 ± 00:04	00:11 ± 00:03	00:15 ± 00:04	00:15 ± 00:03
Sleep efficiency (%)	81.40 ± 1.03	84.23 ± 1.69	82.94 ± 1.08	82.06 ± 1.56
PSQI (0–21)				
Global score	3.39 ± 0.29	4.47 ± 0.39	3.84 ± 0.41	3.86 ± 0.28

Average values ± SEM for actigraphy and PSQI measures for both task and age groups. Values for actigraphy are reported in hours/percentages as indicated. A global PSQI score greater than 5 suggests poor sleep (Buysse et al., 1989). Although none of the participants here fell under the category of poor sleepers, the older age groups showed increased scores relative to the younger participants. Key: PSQI, Pittsburgh Sleep Quality Index.
