# Supplementary material for: Sleep-dependent motor memory consolidation in older adults depends on task demands
Source: Neurobiol Aging. 2015 Mar;36(3):1409–16. doi: 10.1016/j.neurobiolaging.2014.12.014 (PMC4353561; doi:10.1016/j.neurobiolaging.2014.12.014)
Supplement: Supplementary Materials [file mmc1.docx]

Supplementary Materials

Statistical analyses without 9 (repeat) participants

In order to ensure that the inclusion of subjects who participated in more than one experimental condition did not disproportionally influence our results we re-ran the sleep vs wake repeated measures ANOVAs removing the second experimental session for all nine repeat participants, all of whom were in the older participant groups.

**Repeated measures ANOVA across groups:** Results were very similar to the original analyses, with significant main effects of sleep vs wake (F(1,72) = 5.48, p = .022), age group (F(1,72) = 7.80, p = .007). Although the effect of task no longer reached statistical significance (F(1,72) = 2.70, p = .11). These results replicate the overall greater off-line improvements in performance with sleep and in the younger age group. The previously found interactions also held and were here highly significant (sleep vs wake x training time, (F(1,72) = 7.02, p = .01; sleep vs wake x age group x training time, F(1,72) = 8.33, p = .005; sleep vs wake x age group x task x training time, F(1,72) = 7.41, p = .008).

**Repeated measures ANOVA split by task and age group:** Here, results were again very similar to the original findings, and show significant main effects of sleep vs wake in older adults for the adapted (F(1,12) = 8.11, p = .015), but not classic (F(1,7) = .46, p = .52), sequence task. Therefore, we were able to confirm that our conclusions held (i.e., we found evidence for sleep-dependent consolidation in the older groups for the adapted task but a lack of a consolidation effect for the classic task). Note that as all repeat participants were from the older groups there is no need to re-run any statistics concerning the younger groups alone.

We also re-ran the correlation between learning during the initial training session and off-line consolidation effects: Again, results revealed a highly significant relationship across groups even without the second session from the nine repeat participants (r = -.37, p < .001), confirming that the more on-line learning that took place during training, the less off-line consolidation occurred with sleep. No correlation was found between change in-session and after an equivalent period of wakefulness (r = .095, p = .40), and here correlations again differed significantly using Fisher’s r-to-z transformation (z = -3, p = .003).
